# Estradiol (*E*_2_) Reduction Adversely Affect the Embryo Quality and Clinical Outcomes of In Vitro Fertilization and Embryo transfer (IVF-ET)

**DOI:** 10.1155/2022/2473876

**Published:** 2022-04-07

**Authors:** Jing Cheng, Shuangqing Yang, Huaqing Ma, Yingxiu Liang, Junzhao Zhao

**Affiliations:** Reproductive Center, Department of Obstetrics and Gynecology, The Second Affiliated Hospital and Yuying Children's Hospital of Wenzhou Medical University, Wenzhou, China

## Abstract

**Objective:**

The purpose of this study was to explore the influence of decreased serum estradiol (*E*_2_) levels during controlled ovarian hyperstimulation (COH) on in vitro fertilization and embryo transfer (IVF).

**Methods:**

The clinical data of 300 IVF-ET cycles with patients were analyzed retrospectively. According to the presence of falling *E*_2_ level during the COH, we divided all subjects into two groups: the *E*_2_ levels fall group (*n* = 120, group A) and the control group (*n* = 180, group B). In group A, there were 57 patients with falling *E*_2_ with drug dosage reduction. The other 63 patients experienced the decreased *E*_2_ level spontaneously. The clinical and laboratory variables in the groups were compared. Receiver operator characteristic (ROC) curve analyses were carried out in order to evaluate the predict value of *E*_2_ level on the day of human chorionic gonadotropin (hCG) administration on IVF outcomes.

**Results:**

Duration and total dosage of gonadotropin (Gn) used were statistically more in group A than in group B (*P* < 0.001). The high-quality embryo rate was significantly lower in group A (*P* = 0.048). Women in group A had lower clinical pregnancy rate (*P* = 0.029), live birth rate (*P* < 0.001), ongoing pregnancy rate (*P* = 0.001), and higher early abortion rates (*P* = 0.008) than group B. Women with spontaneously falling *E*_2_ group had a higher BMI index than those in the drug dosage reduction group (*P* = 0.001). More dosage and longer duration of Gn in spontaneously falling *E*_2_ group than in the drug dosage reduction group (*P* < 0.01). There were no differences in clinical outcomes between the two types of *E*_2_ decreased groups. Results from ROC showed an *E*_2_ level <1987.5 pg/ml on the hCG day might predict early abortion in this study. The sensitivity was 58.4% and the specificity was 78.9%. In addition, an *E*_2_ level >2020 pg/ml on the hCG day might be an index to predict live birth. The sensitivity was 57.0% and the specificity was 61.7%.

**Conclusions:**

Reduction of *E*_2_ during COH might adversely affect the clinical pregnancy, early abortion, and ongoing pregnancy of IVF-ET.

## 1. Introduction

Infertility is a global condition which affects up to 8–12% of couples in their reproductive age [1]. In vitro fertilization and embryo transfer (IVF-ET) have been widely performed since 1978 for infertile couples.

Controlled ovarian hyperstimulation (COH) is clearly vital in the course of IVF-ET. During the process of COH often requires clinical monitoring, mainly including ultrasound detection and hormonal determination.

The measurement of serum estradiol (*E*_2_) is an important tool used to monitor follicle growth during COH. Preovulatory follicle development during COH depends on an estrogen-dependent environment. The pattern of *E*_2_ level rise is a useful tool to identify poor responders [2]. Typically, serum *E*_2_ levels are gradually elevated prior to human chorionic gonadotropin administration. However, some researchers found that some patients had a decrease in serum *E*_2_ levels before the hCG injection [3, 4]. Some patients experienced falling *E*_2_ levels with drug adjustment during COH. Others experienced the reduction of *E*_2_ levels spontaneously.

Whether the decrease of *E*_2_ levels during COH affects pregnancy outcomes is contradictory in clinic. Fisher et al. reported that the effect of decreased *E*_2_ levels on clinical outcomes was related to the reasons of decreased *E*_2_ levels [5]. Spontaneously falling *E*_2_ was companied with fewer oocytes retrieved and lower clinical pregnancy rates. Their results also pointed out that reduction of *E*_2_ with drug adjustment did not have a noteworthy effect on IVF outcome. In addition, results from Leonti et al. showed the spontaneous decrease of *E*_2_ levels adversely affected IVF outcomes. Furthermore, the percentage decrease of *E*_2_ had a linear correlation with the number of oocytes collected [6]. However, Styer et al. reported that the drop in serum *E*_2_ levels during COH has no effect on clinical outcomes in GnRH-a protocol IVF cycles [7].

The *E*_2_ levels on the hCG day are also an important consideration for clinical outcomes in the clinic. Taşkin et al. retrospectively analyzed the clinical data of 350 fresh cycles with GnRH antagonist or GnRH agonist protocols [8]. They found the serum *E*_2_ level on the hCG day did not affect the IVF clinical outcomes. However, results from Shao et al. showed lower *E*_2_ levels in serum on the hCG day had adverse effects on clinical outcomes in IVF-ET. It was mainly reflected in the impacts on the rate of implantation, the number of oocytes fertilized and retrieved [9].

Up to now, there has been no clear conclusion about the relationship between the decrease of *E*_2_ levels during COH or the *E*_2_ levels on hCG day and the clinical outcomes of IVF. In this study, we retrospectively analyzed clinical data of 300 IVF cycles in our center. The objective of this study was to investigate the influence of decreased *E*_2_ levels during COH on the outcome of IVF with a downregulated protocol.

## 2. Methods

### 2.1. Study Subjects

In this retrospective study, all patients who underwent IVF or intracytoplasmic sperm injection (ICSI) cycles with fresh embryos transferred from June 2017 to December 2019 at the Reproductive Center of the Second Affiliated Hospital and Yuying Children's Hospital of Wenzhou Medical University were included. This study was approved by the Medical Ethics Committee of the Second Affiliated Hospital and Yuying Children's Hospital of Wenzhou Medical University (NO.2021-K-382-01).

**Figure 1:**
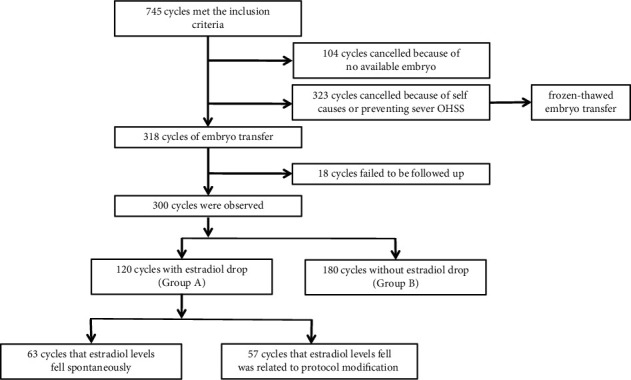
Flow chart of cycles included in the analysis.

745 cycles met the study criteria. 427 cycles were cancelled, including 104 cycles with no available embryo and 323 cycles for personal reasons or preventing ovarian hyperstimulation syndrome (OHSS). All embryos of cancelled cycle were frozen for frozen-thawed embryo transfer. 18 cycles failed to be followed up. Therefore, 300 cycles were finally observed (Figure 1).

The inclusion criteria were as follows: (I) patients with infertility aged ≥20 and ≤40 years old; (II) patients who were undergoing their first cycle. (III) women with menstrual cycle of 25–35 days (IV) all women treated with gonadotropin-releasing hormone agonist protocol; (V) both ovaries intact, and normal renal, liver, and hematological indices; and (VI) no participant had received interventional medication or surgery within the three months before enter the IVF course.

Subjects were excluded with any of the following: (I) with a body mass index (BMI) > 30 kg/m^2^; (II) with endometriosis III/IV stage or adenomyosis; (III) with a malformed uterus, intrauterine adhesion, endometrial polys, or submucous myoma; and (IV) combined with severe male factors (azoospermia or high DNA damage spermatozoa).

### 2.2. COH Protocols

Patients in this study were treated with one of the following protocols: (I) the long protocol; (II) the improved prolonged protocol in the early follicular phase; and (III) the improved prolonged protocol in the midluteal phase.

In the long protocol, patients received 1.875 mg of GnRH-a (triptorelin, Ferring, Kiel, Germany) via an intramuscular injection in the midluteal phase of their menstrual cycles for pituitary down-regulation. Downregulation was confirmed after 14 days and was followed by COH. In the improved prolonged protocol in the early follicular phase, women were treated with 3.75 mg of GnRH-a during days 2–5 of their menstruation cycle. As for the improved prolonged protocol in the midluteal phase, women were injected with 3.75 mg of GnRH-a in the midluteal phase. For all the women with improved prolonged protocols, ovarian stimulation started 30–37 days later from downregulation. Successful pituitary downregulation was confirmed if they met the following criteria: serum luteinizing hormone (LH) level ≤5 IU/L, serum *E*_2_ level ≤50 pg/mL, ovarian follicle diameter ≤5 mm, and endometrial thickness ≤5 mm. Once successful donwregulation was achieved, the start dosage of gonadotropin (Gn) for COH ranged from 75 to 300 IU on the basis of follicle stimulating hormone (FSH) level, body mass index (BMI), and antral follicular count (AFC). The growth of the ovarian follicular was monitored by serum progesterone levels, *E*_2_ levels, LH levels, and transvaginal ultrasonography. The Gn dosage was adjusted with ovarian response. When the diameter of one dominant follicle reached 18 mm or two dominant follicles reached 17 mm, a 5,000–10,000 IU dose of human chorionic gonadotropin (hCG) (Livzon, Guangdong, China) would be administered by intramuscular injection to trigger the final maturation of the follicles.

### 2.3. IVF/ICSI Procedure

Oocyte retrieval was implemented under the guidance of transvaginal ultrasound 36–38 h after hCG administration. 4–6 hours later, oocytes were inseminated through IVF or ICSI. All embryos were incubated under a humidified gas mixture of 90% N_2_, 4% O_2_, and 6% CO_2_ at 37°C. According to the cell numbers of the inner cell mass and the degree of fragmentation on day 3, embryos were graded by a specific professional embryologist. Embryos of Veeck grade one or two were graded as good quality [10]. Embryo transfer (ET) was performed with one or two embryos on the 4∼5 of embryo culture with the assistance of abdominal ultrasound.

Luteal support started from the second day of oocyte collection. Patients were given 20 mg of oral dydrogesterone (Duphaston, Mylan Medical, France) and 90 mg of progesterone vaginal gel for prolonged release per day (Crinone, Merck Serono, United Kingdom) for 15 days. Serum hCG was measured on days 10–14 after ET. Ultrasonography of the pelvis was performed at gestational week 7. Follow-up was not finished until the baby's birth.

### 2.4. Subgroup

All subjects were divided into two groups according to with or without falling *E*_2_ levels during the COH. Group A contained 120 infertile patients whose *E*_2_ levels fell during the COH. Among them, 57 women experienced falling *E*_2_ with drug dosage adjustment. 63 patients endured the *E*_2_ decrease spontaneously. Group B included 180 patients whose *E*_2_ levels during COH did not decrease until hCG administration.

### 2.5. Outcomes

Outcomes measured in this study included clinical pregnancy rates, early abortion rates, ongoing pregnancy rates, and live birth rates. The duration and total dose of Gn administered, number of oocytes collected, rate of available blastocysts, and good quality embryos were evaluated as well. Clinical pregnancy was defined as the detection of an intrauterine gestational sac and fetal heart at 4 weeks after ET through transvaginal ultrasound. Early abortion is defined as a clinical pregnancy unable to reach the 12^th^ week of gestation. Live birth was defined as the delivery of a live child or children after the 28^th^ gestational week.

### 2.6. Statistical Analysis

All the data in this study were analyzed using SPSS 25.0 (USA). The continuous variables were analyzed using analyses of the Mann–Whitney test or the independent samples *t*-test. The categorical variables were assessed using a Chi-square test. Receiver operator characteristic (ROC) curve was to analyse the predict value of serum *E*_2_ level on the hCG day in pregnancy outcomes including an early abortion and a live birth. *P* < 0.05 was considered statistically significant.

## 3. Results

### 3.1. Comparison of the Basic Characteristics, Stimulation Variables, and Clinical Outcomes in Women with *E*_2_ Reduction and without *E*_2_ Reduction

There were no differences in age between group A and group B (*P* = 0.407, Table 1). The BMI of women in group A was significantly higher than in group B (*P* = 0.013; Table 1). Women in group A had more AFC compared with those in group B (*P* < 0.001; Table 1). Most cycles were from an improved prolonged protocol in the follicular phase (IPF). It counted for 80.83% in group A, more than 65.55% in group B (*P* = 0.004; Table 1). However, women with long protocols got an *E*_2_ reduction in group A significantly lower than those in group B (7.50% *vs* 21.67%, *P* = 0.001; Table 1). The chance occurred *E*_2_ decrease or not in COH was similar in improved prolonged protocol in the midluteal phase (11.67% vs 12.78%, *P* = 0.774; Table 1).

**Figure 2:**
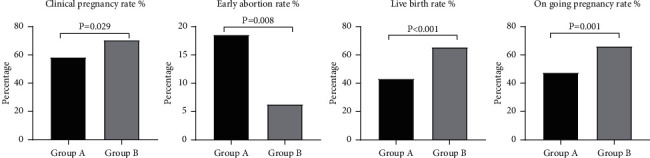
Comparation of clinical index between group A and group B.

During COH, the starting dosage of Gn applied in women from group A was fewer than that from group B (*P* = 0.016; Table 2). Furthermore, duration and total dosage of Gn administered in group A were significantly more than group B (*P* < 0.001, *P* < 0.001, respectively; Table 2). As for hormones on hCG day, levels of *E*_2_ and LH in group A were statistically lower than those in group B (*P* = 0.027, *P* = 0.0045, respectively; Table 2). However, no similar results were found in the P level on hCG day (*P* = 0.429; Table 2). After oocytes were inseminated, the percent of high-quality embryos acquired was statistically lower in group A (*P* = 0.048; Table 2). Consistent with this, the clinical pregnancy rate (58.33% vs 70.56% *P* = 0.029; Table 2 and Figure 2), on-going pregnancy rate (47.50% *vs* 66.11% *P* = 0.001; Table 2 and Figure 2), and live birth rate in group A were significantly lower than those from group B (*P* < 0.001; Table 2 and Figure 2). In addition, the early abortion rate was higher in group A than in group B (*P* = 0.008; Table 2 and Figure 2).

There was no difference in endometrial thickness on the hCG day, numbers of oocytes collected, and available blastocyst rates between the two groups (Table 2).

### 3.2. Comparison of Basic Characteristics, Stimulation Variables, and Clinical Outcomes in Women with *E*_2_ Reduction from Different Causes

According to the causes for *E*_2_ reduction, women in group A was divided into two groups, spontaneously falling *E*_2_ group and medication dosage adjustment group. Women from the spontaneously falling *E*_2_ group had higher BMI compared with those in the medication dosage adjustment group (*P* = 0.001; Table 3). The AFC and age were similar in both two groups (*P* > 0.05; Table 3). Women in the spontaneously falling *E*_2_ group experienced a longer duration of Gn and more dosage than those in the dosage adjustment group (*P* < 0.001 and *P* < 0.001; Table 4). The *E*_2_ levels on the hCG day were lower in the spontaneously falling *E*_2_ group (*P* < 0.01; Table 4). No significant differences were found in LH and P levels on hCG day between the two groups (*P* = 0.744 and *P* = 0.284; Table 4). Patients in the spontaneously falling *E*_2_ group had significantly fewer numbers of oocytes retrieved than those in the drug adjustment group (*P* = 0.001; Table 4). There was no difference in high-quality embryos and blastocyst formation rates, as well as clinical outcomes including clinical pregnancy rates, early abortion rates, ongoing pregnancy rates, and live birth rates between the two causes of *E*_2_ decrease groups (*P* > 0.05; Table 4). Among the long protocols, spontaneous E2 reduction occurred more often than the dose adjustment group (*P* = 0.034; Table 3).

### 3.3. ROC Curve Analysis of the Predictive Value of *E*_2_ Level on hCG Day on the Clinical Outcome of In Vitro Fertilization

**Figure 3:**
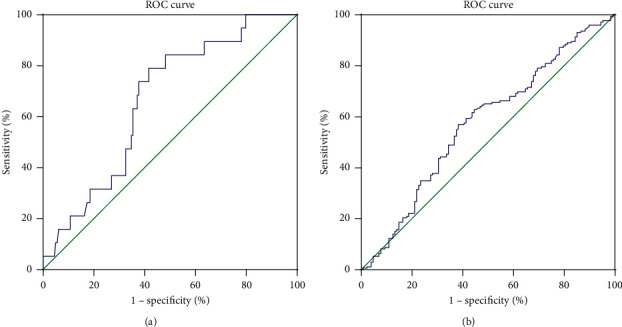
ROC analysis for clinical outcomes. (a) The ability of estradiol level on an hCG day to predict early abortion. The AUC was 0.663, and the optimal estradiol cut-off value was 1987.5 pg/mL. (b) The ability of estradiol level on an hCG day to predict early abortion. The AUC was 0.573, and the optimal estradiol cut-off value was 2020 pg/mL. ROC: receiver operating characteristic.

In this study, ROC curve analysis was used to verify the predictive value of *E*_2_ level on the hCG day on clinical outcomes in IVF. The result of the ROC curve showed that *E*_2_ level on the hCG day might predict early abortion and the AUC of the ROC curve was 0.663. The optimal *E*_2_ level cut-off value was 1987.5 pg/ml with a sensitivity of 78.9% and a specificity of 58.4% (Figure 3). This suggested that patients were more likely to experience early abortion when the *E*_2_ level was lower than 1987.5 pg/ml. In addition, the *E*_2_ level on the hCG day might have the ability for estimating the chance of a live birth. The AUC was 0.573 and the optimal *E*_2_ cut-off value was 2020 pg/ml. The sensitivity was 57.0% and the specificity was 61.7% (Figure 3). That means an *E*_2_ level on the hCG day more than 2020 pg/ml might signify a chance to have a live birth. The predictive ability of the *E*_2_ level to predict other clinical outcomes was not found.

## 4. Discussion

The results of this retrospective study showed that the decrease of *E*_2_ levels during COH might have adversely affected clinical pregnancy rates, accompanied with increasing early abortion rates and reducing ongoing pregnancy rates and live birth rates in GnRH-a protocols. This detrimental impact of *E*_2_ reduction on pregnancy outcomes was not depend on the reasons whether it was induced spontaneously or from drug adjustment. Furthermore, the result of ROC curve analysis showed the *E*_2_ level on the hCG administration day might have the virtue to predict early abortion. Women with *E*_2_ levels lower than 1987.5 pg/ml on hCG day might be associated with early abortion in IVF courses. Thirdly, the results of this study suggested the optimal *E*_2_ level was over 2020 pg/ml to imply a great opportunity to have a live birth.

It has been reported that the decline in serum *E*_2_ levels during GnRH-a downregulated COH was irrelevant to clinical pregnancy outcomes in IVF [7]. However, results from the Fisher group showed the decrease of *E*_2_ level was in connection with low clinical pregnancy rates [5]. Results of this study showed *E*_2_ decline during COH was closely associated with lower clinical pregnancy rates in the downregulation protocol in IVF. Controversial conclusion about the decline of *E*_2_ on clinical pregnancy rate might attribute to the differences in stimulation protocols. Furthermore, ethnic differences might be another reason. The patients enrolled previously were Caucasians [5, 7]. And the patients in our study were all Chinese.

Whenever it comes to clinical pregnancy rate in IVF, endometrial receptivity could not be ignored, as endocrine correlates to markers of endometrial receptivity [11]. It has been reported that an *E*_2_ level higher than 20,000 pmol/L during COH might impair endometrial receptivity in IVF cycles [12]. As far as we know, few studies have investigated the relationship between endometrial receptivity and the sudden decrease in *E*_2_ level during COH. In the clinic, endometrial pattern and thickness are two common indexes to reflect endometrial receptivity. Leonti et al. pointed out, spontaneously decreasing estradiol levels have no effect on endometrial thickness [6]. Similarly, our results showed that no significant differences were found in endometrial thickness on hCG day between the *E*_2_ decline group and the control group. Unfortunately, we failed to get the whole information about the endometrial pattern on the hCG day between groups. It is worth noting that the endometrial receptivity is affected by the dosage of Gn. Gradually increasing doses of Gn during COH affects endometrial receptivity, and influences pregnancy rate [13, 14]. In this study, duration and total dosage of Gn used in the *E*_2_ decline group notwithstanding, thickness of endometrium and level of *E*_2_ on hCG day were no different to the control group. We supposed that lower clinical pregnancy rates and higher early abortion rates might be uncorrelated to the endometrium receptivity.

Patients in the *E*_2_ decline group were administered more Gn doses which might relative to the BMI.

Another important factor influencing clinical pregnancy in IVF is embryo quality. The decrease of *E*_2_ level might be related to embryo quality and further affect pregnancy outcomes in IVF [15, 16]. A recent study explored the effect of a sudden fall of serum *E*_2_ level in donors during ovarian stimulation on recipient outcome [15], which excluded the influence of endometrial receptivity. The results showed a fall in serum *E*_2_ level of more than 30% in donors during ovarian stimulation negatively affected embryo quality in recipients. In this study, we further confirmed that *E*_2_ reduction caused a detriment effect on embryos, leading in fewer high-quality embryos. These results in turn contributed to the lower clinical pregnancy rates, ongoing pregnancy rates, and higher early abortion rates in the *E*_2_ decline group.

Embryo quality is closely related to follicular function and oocyte health. Recently, one study reported that oocytes might be impaired when exposed to a high FSH level [17]. Possibly, spindle morphology is damaged and chromosome congression is caused [17, 18]. Another retrospective study found that fewer Gn doses in COH were a positive independent predictor of clinical pregnancy rate [19]. In the present study, the *E*_2_ decline group had a longer duration and a higher dosage of Gn used compared with the control group. We supposed that the increase of Gn in the *E*_2_ decline group might impact oocyte quality, influence embryo development and lead to adverse pregnancy outcomes.

Obesity has been recognized as another detriment factor to pregnancy rates in IVF. Higher BMI might reduce the response of granulosa cells to FSH stimulation, resulting in a decrease in *E*_2_ production [20]. In addition, obese women had an increased risk of abortion [21]. Consistent with these results, we found the women in the falling *E*_2_ group had a higher BMI. Thus, it was reasonable to suppose that lower clinical pregnancy rates and higher early abortion rates in the *E*_2_ decline group might partly result from higher BMI.

Clinically, the decreased *E*_2_ levels during COH might be due to spontaneous or drug dosage adjustment. One study reported that *E*_2_ reduction from dose-modification has no effect on the clinical pregnancy rate, and that only a spontaneous E2 decrease cause an adverse effect on clinical pregnancy rates [5]. Differing from previous study, results from our study suggested that once *E*_2_ declined occur during COH, no matter whether spontaneously *E*_2_ falling or drug modification, it would had adverse effect on clinical outcomes in IVF women. The reason for the different results might be the difference in timing of Gn reduction. At different stages of follicular development, follicles have different thresholds for FSH [22]. We generally adjusted the Gn dose when more follicles with a diameter of 14 mm or the *E*_2_ level in serum higher than 4000 pg/ml.

*E*_2_ levels on the day of hCG administration might has been report affects pregnancy outcomes in IVF cycles. It can be concentration-dependent style and influence the pregnancy outcome [23]. The serum *E*_2_ level on the hCG day in patients varied with age in different studies [23, 24]. In the present study, we found women with an *E*_2_ level lower than 1987.5 pg/ml on the hCG day might be an added risk to the early abortion. Besides, the *E*_2_ level on the hCG day higher than 2020 pg/ml might imply more chance to have a live birth. These results give us a scope of *E*_2_ on hCG day that we should reach during COH, which is more helpful to improve the clinical outcomes in IVF.

There were limitations in our study. Firstly, the sample size in the study was relatively small. Because of the limitation of sample size, we were unable to explore the difference in the effect of decreased *E*_2_ levels on pregnancy outcomes among the three protocols. Also, because of the limitation of small sample, we were unable to investigate the correlation between IVF outcomes and the degree of *E*_2_ level decline. In future studies, further expanding the sample size is needed to explore the impact of decreased *E*_2_ levels on IVF outcomes with different COH protocols. It would be helpful for clinicians to choose the optimal protocols.

## 5. Conclusion

In summary, the decrease of serum *E*_2_ levels during COH might decrease the clinical pregnancy rate, increase early abortion rates, and reduce the live birth rate in IVF patients. These detrimental effects of *E*_2_ decline on IVF outcomes are not correlated to the decline reasons. In addition, if the *E*_2_ level is lower than 1987.5 pg/ml on the hCG day, then there is a risk for early abortion, and if it is higher than 2020 pg/ml, then might be a live birth. All these results are only for patients with GnRH agonist protocols in IVF.

## Figures and Tables

**Table 1:** Comparison of basic characteristics between group A and group B.

Variables	Group A (*n* = 120)	Group B (*n* = 180)	*P* value
Age (years)	30.36 ± 4.13	30.00 ± 3.21	0.407
BMI (kg/m^2^)	22.04 ± 3.46	21.01 ± 3.48	0.013
*Protocol*			
Long % (*n*)	7.50% (9/120)	21.67% (39/180)	0.001
IPM % (*n*)	11.67% (14/120)	12.78% (23/180)	0.774
IPF % (*n*)	80.83% (97/120)	65.55% (118/180)	0.004
AFC (*n*)	21.74 ± 9.95	16.75 ± 7.09	<0.001

BMI: body mass index' Long: long protocol; IPM: improved prolonged protocol in the midluteal phase; IPF: improved prolonged protocol in the follicular phase; AFC: antral follicle counts.

**Table 2:** Comparison of the stimulation variables and clinical outcomes between group A and group B.

Variables	Group A (*n* = 120)	Group B (*n* = 180)	*P* value
Starting dosage of Gn (IU)	165.02 ± 53.32	180.59 ± 55.32	0.016
Duration of Gn (d)	14.95 ± 3.95	11.21 ± 1.98	<0.001
Total dosage of Gn (IU)	2721.19 ± 1291.25	2258.64 ± 876.05	<0.001
Endometrial thickness on the hCG day (mm)	11.09 ± 2.17	11.14 ± 1.97	0.850
LH level on the hCG day (IU/L)	0.57 ± 0.49	0.75 ± 0.58	0.005
*E*_2_ level on the hCG day (pg/ml)	1906.76 ± 1190.35	2203.77 ± 1032.71	0.027
P level on the hCG day (ng/mL)	0.57 ± 0.30	0.59 ± 0.28	0.429
Number of oocytes retrieved (*n*)	12.89 ± 5.76	12.09 ± 5.60	0.231
Rate of high-quality embryos (%)	0.79 ± 0.23	0.84 ± 0.20	0.048
Blastocysts available rate (%)	0.45 ± 0.30	0.46 ± 0.28	0.801
Clinical pregnancy rate (%)	58.33% (70/120)	70.56% (127/180)	0.029
Early abortion rate (%)	18.57% (13/70)	6.30% (8/127)	0.008
Ongoing pregnancy rate (%)	47.50% (57/120)	66.11% (119/180)	0.001
Live birth rate (%)	43.33% (52/120)	65.56% (118/180)	<0.001

hCG, human chorionic gonadotropin; Gn, gonadotropin.

**Table 3:** Comparison of basic characteristics between the spontaneously falling *E*_2_ group and the drug dosage reduction group.

Variables	Spontaneously reduction (*n* = 63)	Dose-adjusted reduction (*n* = 57)	*P* value
Age (years)	30.67 ± 4.18	30.02 ± 4.09	0.392
BMI (kg/m^2^)	23.04 ± 3.62	20.93 ± 2.92	0.001
AFC (*n*)	22.42 ± 10.35	21.06 ± 9.59	0.497
*Protocol*			
Long % (*n*)	12.70% (8/63)	1.75% (1/57)	0.034
IPM% (*n*)	7.94% (5/63)	15.79% (9/57)	0.181
IPF% (*n*)	79.36 (50/63)	82.46% (47/57)	0.667

BMI, body mass index. Long: long protocol; IPM: improved prolonged protocol in the midluteal phase; IPF: improved prolonged protocol in the follicular phase; AFC: antral follicle counts.

**Table 4:** Comparison of the stimulation variables and clinical outcomes between the spontaneously falling E2 group and the drug dosage reduction group.

Variables	Spontaneously reduction (*n* = 63)	Dose-adjusted reduction (*n* = 57)	*P* value
Starting dosage of Gn used (IU)	156.55 ± 49.23	174.55 ± 56.51	0.068
Duration of Gn used (d)	16.38 ± 4.38	13.37 ± 2.66	<0.001
Total dosage of Gn used (IU)	3147.22 ± 1448.74	2250.32 ± 888.53	<0.001
Endometrial thickness on hCG day (mm)	11.07 ± 2.41	11.11 ± 1.90	0.920
LH level on the hCG day (IU/L)	0.59 ± 0.58	0.56 ± 0.36	0.744
*E*_2_ level on the hCG day (pg/ml)	1517.31 ± 936.48	2330.37 ± 1296.09	<0.001
P level on the hCG day (ng/mL)	0.54 ± 0.30	0.60 ± 0.31	0.284
Number of oocytes retrieved (*n*)	11.19 ± 5.50	14.77 ± 5.46	0.001
Rate of high-quality embryos (%)	0.77 ± 0.26	0.81 ± 0.20	0.299
Blastocysts available rate (%)	0.40 ± 0.30	0.51 ± 0.28	0.053
Clinical pregnancy rate (%)	61.90% (39/63)	54.39% (31/57)	0.404
Early abortion rate (%)	17.95% (7/39)	19.35% (6/31)	0.881
Ongoing pregnancy rate (%)	50.79% (32/63)	43.86% (25/57)	0.448
Live birth rate (%)	47.62% (30/63)	38.60% (22/57)	0.319

hCG, human chorionic gonadotropin; Gn, gonadotropin.

## Data Availability

The data used to support the findings of this study are available from the corresponding author upon request.
